# Xanthine-Catechin Mixture Enhances Lithium-Induced Anti-Inflammatory Response in Activated Macrophages* In Vitro*

**DOI:** 10.1155/2017/4151594

**Published:** 2017-11-08

**Authors:** Fernanda Barbisan, Verônica Farina Azzolin, Cibele Ferreira Teixeira, Moisés Henrique Mastella, Euler Esteves Ribeiro, Pedro Antonio Schmidt do Prado-Lima, Raquel de Souza Praia, Marta Maria Medeiros Frescura Duarte, Ivana Beatrice Mânica da Cruz

**Affiliations:** ^1^Postgraduate Program of Pharmacology, Federal University of Santa Maria, Santa Maria, RS, Brazil; ^2^Postgraduate Program of Gerontology, Federal University of Santa Maria, Santa Maria, RS, Brazil; ^3^Open University of the Third Age, State University of Amazonas, Manaus, AM, Brazil; ^4^Brain Institute, Catholic University of Rio Grande do Sul, Porto Alegre, RS, Brazil; ^5^Lutheran University of Brazil, Santa Maria, RS, Brazil

## Abstract

Lithium (Li) is a chemical element used for treating and preventing bipolar disorder (BD) and exerts positive effects such as anti-inflammatory effects as well as undesirable side effects. These effects of Li can be influenced by interaction with some nutritional elements. Therefore, we investigated the potential effects of xanthine (caffeine and theobromine) and catechin molecules present in some food beverages broadly consumed worldwide, such as coffee and tea, on Li-induced anti-inflammatory effects. In the present study, we concomitantly exposed RAW 264.7 macrophages to Li, isolated xanthine and catechin molecules, and a xanthine-catechin mixture (XC mixture). We evaluated the effects of these treatments on cell proliferation, cell cycle progression, oxidative and antioxidant marker expression, cytokine levels, gene expression, and GSK-3*β* enzyme expression. Treatment with the XC mixture potentialized Li-induced anti-inflammatory effects by intensification of the following: GSK-3*β* inhibitory action, lowering effect on proinflammatory cytokines (IL-1*β*, IL-6, and TNF*α*), and increase in the levels of IL-10 that is an anti-inflammatory cytokine. Despite the controversial nature of caffeine consumption by BD patients, these results suggested that consumption of caffeine, in low concentrations, mixed with other bioactive molecules along with Li may be safe.

## 1. Introduction

Lithium (Li) is a chemical element that naturally occurs in soil and water (mostly at low concentrations) and enters the food chain. Low levels of Li are suggested to exert beneficial effects, and high levels of Li are suggested to exert toxic effects in living organisms [1]. Li has been used for treating psychiatric disorders for over 50 years and continues to be a first-line drug for treating and preventing the symptoms of bipolar disorder (BD) [2].

Pharmacologically, Li exerts different effects, including anti-inflammatory effects, on various biological and biochemical pathways, unlike conventional drugs that interact with specific molecular targets. Li inhibits the expression of glycogen synthase kinase 3 (GSK3), a proinflammatory enzyme that is directly implicated in the pathogenesis of mood disorders such as BD (Luca et al. [3]).

Despite its recognized efficacy, Li has a poor safety profile because of its narrow therapeutic window. Therefore, Li concentrations and clinical symptoms of patients treated with psychiatric drugs should be monitored regularly [4]. Common side effects of Li include gastrointestinal disturbances such as nausea and diarrhea; excessive urination; thirst (polyuria and polydipsia); tremor, primarily of the hands; weight gain; cognitive impairment; and sexual dysfunction, primarily in men and renal insufficiency, which is a serious and potentially fatal side effect [2, 4].

Primary therapeutic strategies used for managing the side effects of Li therapy include use of diuretics for treating polyuria; use of beta-blockers such as primidone, benzodiazepines, or vitamin B6 for treating tremor; exercise and calorie restriction for preventing weight gain; use of stimulants for managing cognitive impairment; and use of aspirin or phosphodiesterase-5 inhibitors for treating sexual dysfunction [4].

However, positive and negative effects of Li can be influenced by interaction with some nutritional elements such as those present in food beverages, including coffee, tea, and guarana (*Paullinia cupana*, mainly used as caffeine source for the industrial production of energy beverages), that are consumed highly and habitually worldwide. These food beverages contain bioactive molecules such xanthine (caffeine and theobromine) and catechins [5]. Moreover, previous studies have suggested that some properties of guarana, including diuretic, thermogenic, energetic, antiobesogenic, neuroprotective, and antidepressant properties, are similar to those of green tea [6–13].

Therefore, in the present study, we determined the potential effect of isolated caffeine and theobromine molecules and of xanthine-catechin (XC) mixture on Li-induced anti-inflammatory effects. The potential interactions between Li and primary bioactive molecules in the XC mixture and their effect on oxidative and inflammatory responses were determined* in vitro* by using RAW 264.7 macrophages, with guarana as a reference.

## 2. Methods

### 2.1. Experimental Design

This* in vitro *study included commercial murine RAW 264.7 macrophages obtained from American Type Culture Collection (Manassas, USA). We selected this experimental model because of the following three reasons. (1) Human genetic polymorphisms directly affect inflammation in human peripheral blood mononuclear cells. For example, Val16Ala-SOD2 single nucleotide polymorphism (rs4880) found in genes encoding manganese-dependent enzymes such as superoxide dismutase (SOD) is associated with differential oxidative and inflammatory responses to drugs and bioactive molecules present in foods [14–20]. (2) Several studies have consistently suggested that psychiatric disorders such as BD are associated with chronic inflammatory conditions [21]. (3) Oxidative stress and inflammation mediate the toxic effects of pharmacological or environmental agents.

The potential interaction between Li and XC molecules (LXC mixture) was determined by evaluating the proliferation and cell cycle progression, oxidative marker (reactive oxygen species [ROS], superoxide anions [SAs], and nitric oxide [NO]) levels, DNA damage, and inflammatory cytokine (interleukin-1*β* [IL-1*β*], IL-6, tumor necrosis factor-alpha [TNF*α*], and IL-10) levels in macrophages. In addition, we evaluated the effect of the LXC mixture on the expression of genes encoding cytokines and GSK-3 enzyme. Phytohemagglutinin (PHA), which triggers inflammation in macrophages, was used as a positive control. All experiments were performed in triplicate.

### 2.2. Cell Culture and Treatments

RAW 264.7 macrophages were cultured in DMEM supplemented with 10% fetal bovine serum (FBS), penicillin (100 U/mL), and streptomycin (100 mg/mL), as described previously by Jung et al. [22]. The cultures were maintained at 37°C under standard humidity and 5% CO_2_ in a standardized, controlled incubator. All experiments were repeated at least three times. Macrophages were treated with the following concentrations of each molecule: 0.17, 0.35, 0.7, 1.4, and 2.80 mEq/L Li; 25, 50, 100, 200, and 400 *μ*g/mL caffeine; 25, 50, 100, 200, and 400 *μ*g/mL theobromine; and 25, 50, 100, 200, and 400 *μ*g/mL catechin. All experiments were performed using Li, caffeine, theobromine, and catechin. Purified molecules were obtained from Sigma-Aldrich (St. Louis, Missouri, USA).

### 2.3. Cell Proliferation Assay

All analyses involving the measurement of absorbance or fluorescence were performed using SpectraMax i3x Multi-Mode Microplate Reader (Molecular Devices, Sunnyvale, California, USA). Cell proliferation was determined by performing 3-(4,5-dimethylthiazol-2-yl)-2,5-diphenyltetrazolium bromide (MTT) reduction spectrophotometric assay, as described previously by Barbisan et al. [20]. Briefly, MTT was dissolved in 5 mg/mL phosphate-buffered saline (PBS) and was added to a 96-well plate containing the sample treatments, and the plate was incubated for 1 h at 37°C. Culture supernatant was removed, and the cells were resuspended in 200 *μ*L of dimethyl sulfoxide (DMSO). Absorbance was read spectrophotometrically at 560 nm.

Effect of the LXC mixture on cell cycle progression was evaluated by performing flow cytometry analysis by exposing the cells to propidium iodide (PI) for 72 h, according to a protocol described by Azzolin et al. [23]. Briefly, the cells were seeded in six-well plates (density, 5 × 10^4^ cells/well) containing 2 mL of the different treatments in DMEM and were incubated for 24 and 72 h. After incubation, the cells were trypsinized, washed with PBS, resuspended in 70% ethanol, and stored overnight at −20°C. Next, the cells were centrifuged, washed once with PBS, and resuspended in 500 *μ*L of PI solution in PBS (50 *μ*g/mL PI from 50x stock solution [2.5 mg/mL], 0.1 mg/mL RNase A, and 0.05% Triton X-100). Finally, the cells were incubated for 40 min at 37°C, washed with 3 mL PBS, and resuspended in 500 *μ*L of PBS for performing flow cytometry analysis.

### 2.4. Quantification of Oxidative Stress Markers and Antioxidant Enzymes

The following oxidative markers were analyzed and compared among cells exposed to the different treatments. NO is crucial for inflammatory response. This molecule was quantified with a colorimetric assay used to detect organic nitrate [14] by the Griess spectrophotometric assay. This assay detects nitrite formed by the spontaneous oxidation of NO under physiological conditions involving azo coupling between diazonium species, which are derived from sulfanilamide and NO_2_, and naphthylethylenediamine. This reaction produces a colorimetric product whose level is proportional to NO present in the sample and can be measured spectrophotometrically at 540 nm [24]. All procedures for determining indirect NO levels were performed at room temperature by using a standard nitrate solution (10 mL) serially diluted in a 96-well plate. Samples were added to the wells, followed by the immediate addition of Griess reagent. Griess reagent was produced by adding equivalent parts of solution 1 containing 1% sulphanilamide diluted in 5% phosphoric acid and solution 2 containing 0.1% N-(1-Naphthyl)ethylenediamine dihydrochloride diluted in 5% phosphoric acid. Linear regression of the mean values of the absorbance at 540 nm for each standard set minus the blank values was used in order to estimate the nitrite or total NO concentrations in samples. These values were then subtracted to obtain nitrate concentration. It is advisable to determine nitrite and total NO concentrations for a particular sample in the same plate to maintain identical conditions for each measurement.

O^2−^ levels were quantified by performing an assay that produced formazan salt through a reaction between nitroblue tetrazolium (NBT) chloride and O^2−^ (Morabito et al. [25]). Briefly, the cells were seeded in a 96-well plate, diluted in 1x PBS, treated with 10 *μ*L of NBT solution (10 mg/mL), homogenized, incubated at 37° for 3 h, and centrifuged. Next, 75 *μ*L of supernatant was removed, and the same volume of DMSO was added to each well. After incubation for 20 min at 37°C, 75 *μ*L of cell suspension was transferred to another 96-well plate, and absorbance was measured at 550 nm.

ROS levels were estimated by performing dichlorofluorescein diacetate (DCFH-DA) fluorometric assay [26]. DCFH-DA (D6883; Sigma-Aldrich) is a nonfluorescent compound that is deacetylated by mitochondrial esterases to 2′,7′-dichlorofluorescein, which reacts with ROS to form a fluorescent compound. To perform this assay Tris-HCl was added in 96-well plate followed by sample and DCFH-DA. The cell samples were homogenized and incubated in the dark at room temperature for 60 min. Fluorescence was measured at an excitation wavelength of 488 nm and an emission wavelength of 525 nm.

Lipoperoxidation was estimated spectrophotometrically by determining the formation of thiobarbituric acid reactive substances, as described previously [27]. Briefly, all reagents, that is, 1x TBA acid diluent, SDS lysis solution, TBA reagent, and 1x BHT solution, were mixed. Each MDA-containing sample and standard were assayed in duplicate. All the reagents and samples were incubated in a water bath at 95°C for 1 h. Next, the samples were cooled and centrifuged at 1000 rpm for 10 min, and absorbance was read at 532 nm.

Protein carbonylation was determined using a method described by Martin et al. (2005) [28]. This is a classical assay involving a reaction between protein carbonyl groups and DNPH, followed by the spectrophotometric quantification of acid hydrazones. For this, the sample was diluted by 1 : 80 by using Tris-HCl buffer; treated with 200 *μ*L DNPH; and incubated in the dark at room temperature for 60 min, with intermittent shaking at 15-min intervals. Next, 500 *μ*L of denaturation buffer (3% SDS), 2000 *μ*L of ethanol, and 2000 *μ*L of hexane were added to the sample, and the sample was shaken for 40 s and centrifuged at 3000 rpm for 15 min. Supernatant obtained was removed, and pellet was resuspended in 1000 *μ*L of denaturation buffer and was incubated in a water bath (40°C–50°C) for 20 min until it dissolved completely. Next, 100 *μ*L of each sample was transferred to a 96-well plate in triplicate, and absorbance was read at 370 nm.

Effect of the different treatments on the levels of endogenous antioxidant enzymes such as SOD, catalase (CAT), and glutathione peroxidase (GPx) was determined using commercial kits specific for each enzyme (Sigma-Aldrich), according to the manufacturer's instructions. GPx-1 activity was measured indirectly by monitoring NADPH oxidation.

### 2.5. Quantification of Cytokine Levels

Levels of cytokines IL-1*β*, IL-6, TNF*α*, and IL-10 in cell culture supernatants were measured using Quantikine Human Kits (R&D Systems, Minneapolis, USA), according to the manufacturer's instructions. Protocol used was similar to that described by Jung et al. [22]. All reagents and working standards were prepared, and excess microplate strips were removed before adding 50 *μ*L of RD1W (assay diluent) to each well. Next, 100 *μ*L of standard control for the samples was added to each well, and the wells were covered with adhesive strips and incubated for 1.5 h at 25°C. Cells in each well were aspirated and washed twice (a total of three washes). Next, antiserum against each molecule to be analyzed was added to each well, and the wells were covered with new adhesive strips and incubated for 30 min at room temperature. The aspiration and washing steps were repeated. The conjugate (100 *μ*L) was added to each well, and the plate was incubated for 30 min at room temperature. The aspiration and washing steps were repeated again. Next, 100 *μ*L of substrate solution was added to each well, and the plate was incubated at room temperature for 20 min. The assay was terminated by adding 50 *μ*L of stop solution to each well, and optical density was determined within 30 min by using a microplate reader set at 450 nm.

### 2.6. Determination of Cytokine and GSK3 Enzyme Gene Expression

Gene expression was determined by performing quantitative reverse transcription-PCR (qRT-PCR) with Rotor-Gene Q 5plex HRM System (QIAGEN Biotechnology, Hilden, North Rhine-Westphalia, Germany). After each treatment, total RNA was extracted using Trizol reagent (Invitrogen Life Technologies, Carlsbad, California, USA). Expression of most genes was quantified, as described previously by Luca et al. [3]. For performing reverse transcription, RNA was added to the samples (1 *μ*L) with 0.2 *μ*L DNase (Invitrogen Life Technologies) for 5 min at 37°C and the samples were heated at 65°C for 10 min. cDNA was synthesized using 1 *μ*L iScript cDNA and 4 *μ*L of Mix iScript (Bio-Rad Laboratories, Hercules, California, USA) by using the following reaction conditions: 5°C for 10 min, 25°C for 5 min, 85°C for 5 min, and 5°C for 60 min.

qRT-PCR was performed using the following reaction conditions: 95°C for 3 min, 40 cycles of 95°C for 10 s and 60°C for 30 s, and a melt curve of 60°C–90°C, with 0.5°C increments, for 5 s. For each sample, qRT-PCR was performed in triplicate by using 1 *μ*M of each primer, 1000 ng/*μ*L cDNA, RNase-free water, and 2x QuantiFast SYBR® Green PCR Master Mix (QIAGEN Biotechnology) in a 20-*μ*L reaction mixture. The housekeeping gene *β*-actin was used as an internal control. Relative expression was quantified using comparative cytosine-thymine (CT) and is expressed as a fold change compared with that in control cells.

Genes encoding cytokines were amplified using the following primers: IL-1*β* forward (F), 5′-GCGGCATCCAGCTACGAAT-3′; reverse (R) 5′-ACCAGCATCTTCCTCAGCTTGT-3′; IL-6: F 5′-TACCCCCAGGAGAAGATTCCA-3′; IL-6; R: 5′-CCGTCGAGGATGTACCGAATT-3′; TNF*α* F: 5′-CAACGGCATGGATCTCAAAGAC-3′; R: 5′-TATGGGCTCATACCAGGGTTTG-3′; IL-10 F: 5′-GTGATGCCCCAAGCTGAGA-3′; R: 5′-TGCTCTTGTTTTCACAGGGAAGA-3′; GSK3-*β* F: 5′-CTCTGGCCACCATCCTTATC-3′; GSK3-*β* R: 5′-CACGGTCTCCAGCATTAGTATC-3′, *β*-actin F: 5′- TGTGGATCAGCAAGCAGGAGTA-3′; R: 5′-TGCGCAAGTTAGGTTTTGTCA-3′.

### 2.7. Statistical Analysis

Statistical analysis was performed using GraphPad Prism 5 software (GraphPad Software, Inc., Brazil). All results are calculated as a percentage of results obtained for negative control group and are expressed as mean ± standard deviation. Results obtained for the different treatments were compared using one-way analysis of variance (ANOVA), followed by Tukey or Bonferroni post hoc test, as appropriate. A *p* value of* <*0.05 was considered statistically significant.

## 3. Results

**Figure 1:**
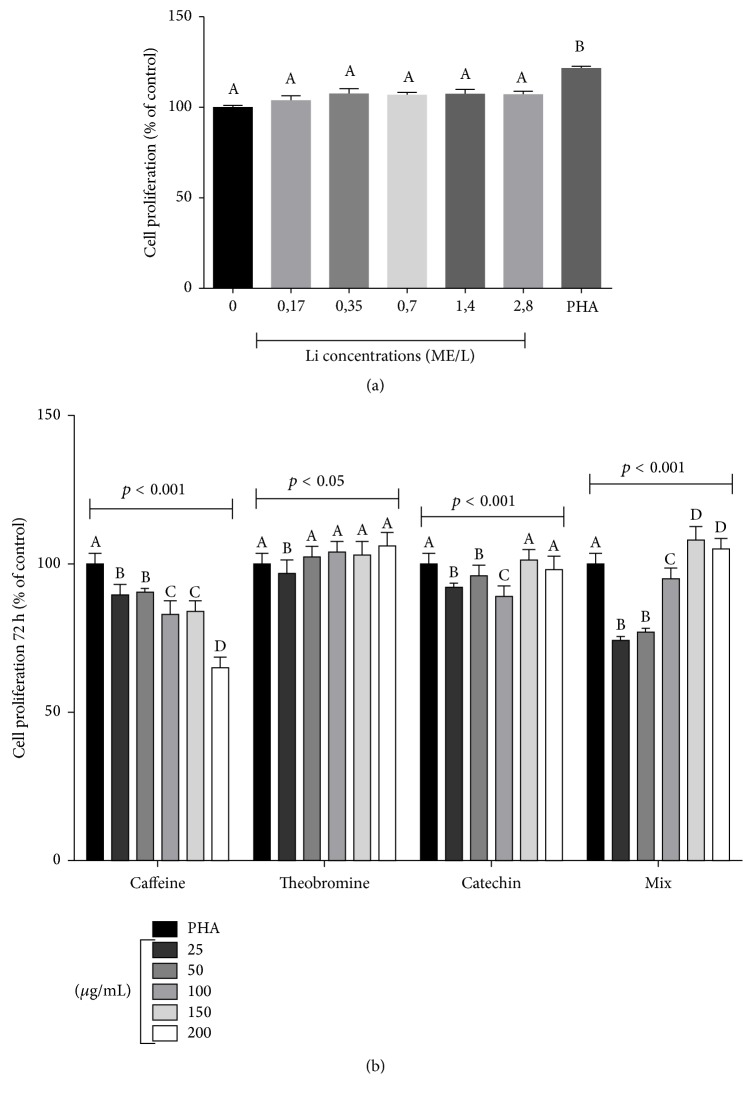
Effect of different concentrations of lithium (Li), isolated xanthine-catechin (XC) molecules, and XC mixture on macrophage proliferation in 72 h cultures. Effect of different Li concentrations on macrophage proliferation was analyzed using one-way analysis of variance (ANOVA), followed by the Tukey post hoc test. Effect of different XC molecule concentrations on macrophage proliferation was analyzed by performing two-way ANOVA followed by the Bonferroni post hoc test. The different letters (i.e., A, B, C, and D) indicate statistical differences in each treatment at *p* < 0.05.

We first analyzed the effect of Li, isolated XC molecules, and XC mixture on macrophage proliferation in 72 h cultures (Figure 1). Li treatment did not affect macrophage proliferation, whereas XC molecule treatment heterogeneously altered macrophage proliferation. Caffeine treatment significantly decreased cell proliferation ratio in a dose-dependent manner.

Treatment with theobromine, except at low concentration (25 *μ*g/mL), did not affect macrophage proliferation. Treatment with low concentrations (25–100 *μ*g/mL) of catechin decreased macrophage proliferation, whereas treatment with high concentrations (>100 *μ*g/mL) of catechin exerted similar effect on macrophage proliferation as the control treatment. Treatment with low concentrations of the XC mixture exerted a decreased effect on macrophage proliferation, which was similar to that observed in cells treated with catechin. However, cell proliferation ratio after treatment with >100 *μ*g/mL XC mixture was higher than that obtained after the control treatment.

Considering that the lower concentration (25 *μ*g/mL) of isolated XC molecules was represented in the 25 *μ*g/mL concentration of XC mixture, the following experiments evaluated the interaction between Li at 0.7 MEq/L, an estimated therapeutic concentration, and XC mixture at a concentration of 25 *μ*g/mL.

**Figure 2:**
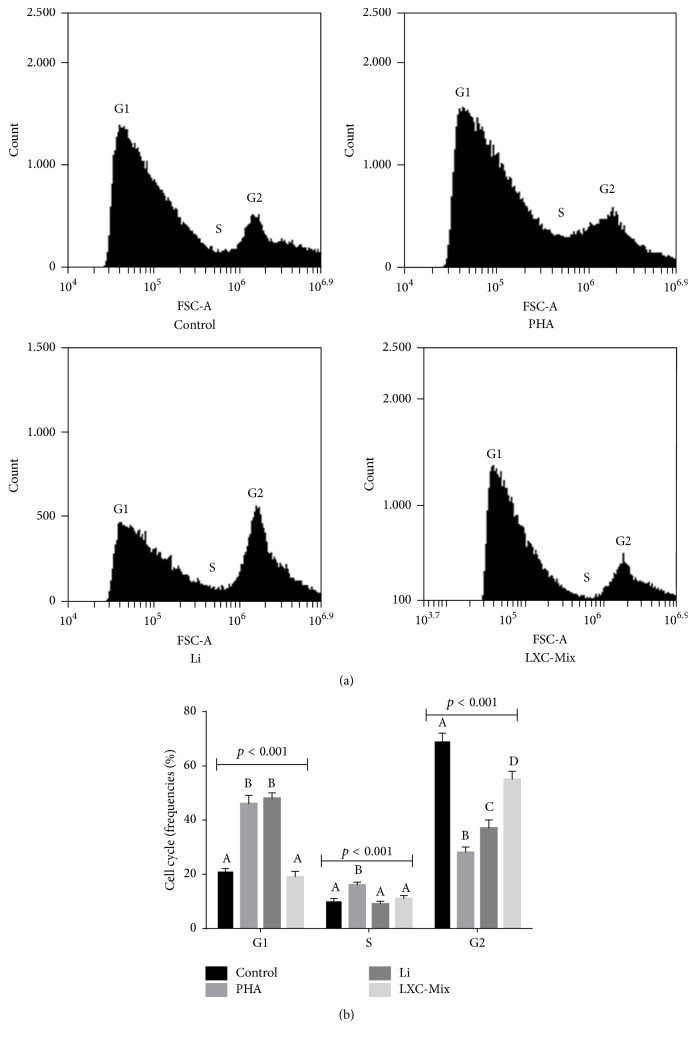
Comparison of cell cycle progression in activated macrophages treated with phytohemagglutinin (PHA), lithium (Li), xanthine-catechin (XC) mixture, and Li and XC (LXC) mixture and cultured for 72 h. (a) Representative flow cytometry analysis graphs for each treatment. G1 = gap 1 phase, G2 = gap 2 phase, and S = synthesis phase. Statistical analysis was performed using two-way analysis of variance followed by Bonferroni post hoc test. The different letters (i.e., A, B, C, and D) indicate statistical differences in each treatment at *p* < 0.05.

Evaluation of the cell cycle in macrophage 72 h cell cultures was directly affected by exposure to PHA, Li, and XC mixture (Figure 2). The proportion of cells in G1 and S phases of the cell cycle was significantly higher among PHA-treated macrophages than among control macrophages.

The increased proportion of PHA-treated macrophages in the G1 phase of the cell cycle may be because flow cytometry analysis includes cells in the G0 phase, which are recently produced by mitosis, in the G1 phase.

The proportion of cells in the G2 phase of the cell cycle was lower among PHA-treated macrophages than among control macrophages. The proportion of cells in the G1 phase was similar between Li-treated macrophages and PHA-treated macrophages, and the proportion of cells in the S phase was similar between Li-treated macrophages and control macrophages. The G2 frequency in Li-activated cells was between that of control and PHA-exposed cells.

The proportion of cells in the G1 and S phases was similar between LXC mixture-treated macrophages and control macrophages. However, the proportion of cells in the G2 phase was higher among LXC mixture-treated macrophages than that among PHA- and Li-treated macrophages but was lower than that among control macrophages. Overall, cell cycle progression in LXC mixture-treated macrophages was similar to that in control macrophages.

**Figure 3:**
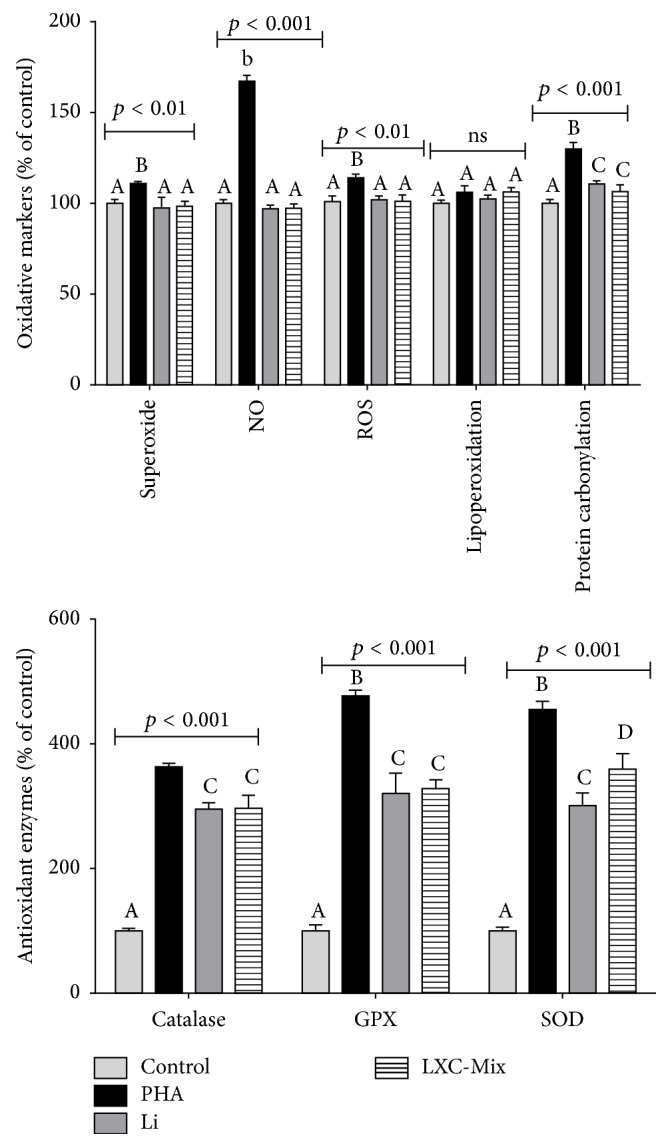
Comparison of changes in the levels of oxidative markers in macrophages treated with phytohemagglutinin (PHA), lithium (Li), xanthine-catechin (XC) mixture, and Li and XC (LXC) mixture and incubated for 72 h. GPx = glutathione peroxidase, NO = nitric oxide, ROS = reactive oxygen species, and SOD = superoxide dismutase. Statistical analysis was performed using two-way analysis of variance followed by the Bonferroni post hoc test. The different letters (i.e., A, B, C, and D) indicate statistical differences in each treatment at *p* < 0.05.

Next, we examined changes in oxidative metabolism in PHA-treated macrophages treated with or without Li and LXC mixture (Figure 3). Macrophages PHA-treated presented higher levels of superoxide, NO, and ROS markers than control macrophages, whereas lipoperoxidation presented similar levels between PHA and control groups. Li and LXC mixture showed similar values to the control group in all markers, except for protein carbonylation. Regarding protein carbonylation, cells Li- and LXC-treated present. Levels of antioxidant enzymes were higher in PHA-treated macrophages than in control macrophages. Although treatment with Li and LXC mixture significantly increased the levels of antioxidant enzymes, this increase was lower than that in PHA-treated macrophages.

**Figure 4:**
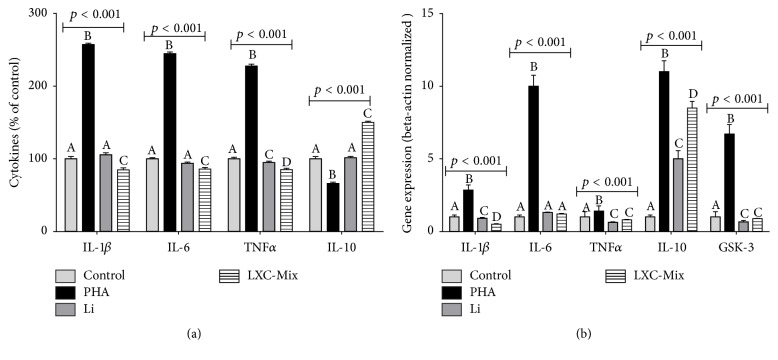
Comparison of cytokine levels (a) and cytokine and GSK-3 gene expression (b) in macrophages treated with phytohemagglutinin (PHA), lithium (Li), xanthine-catechin (XC) mixture, and Li and XC mixture (LXC) and incubated for 72 h. Gene expression in all the samples was normalized to that of the housekeeping gene *β*-actin and is expressed as fold change compared with that in control cells. Statistical analysis was performed using two-way analysis of variance followed by the Bonferroni post hoc test. The different letters (i.e., A, B, C, and D) indicate statistical differences in each treatment at *p* < 0.05.

Next, we investigated the effect of the different treatments on the modulation of cytokine levels and cytokine and GSK-3*β* gene expression (Figure 4). As expected, levels of proinflammatory cytokines IL-1*β*, IL-6, and TNF*α* and GSK-3 enzyme and expression of their corresponding genes were higher in PHA-treated macrophages than in control macrophages. However, level of IL-10 was lower and expression of its corresponding gene was higher in PHA-treated macrophages than in control macrophages. Levels of IL-1*β*, IL-6, and IL-10 were similar in Li-treated and control macrophages. However, TNF*α* levels were lower in Li-treated macrophages than in control macrophages. Li treatment downregulated the expression of all proinflammatory cytokine genes and GSK-3 gene compared with that in control macrophages. Treatment with the LXC mixture decreased the levels of all the proinflammatory cytokines but increased the level of the anti-inflammatory cytokine IL-10. Moreover, treatment with the LXC mixture downregulated the expression of all proinflammatory cytokine genes and GSK-3 gene and upregulated the expression of the IL-10 gene compared with that in control macrophages.

## 4. Discussion

In the present study, we found that Li and Li plus a chemical matrix compound by caffeine, theobromine, and catechin (LXC) are able to modulate oxidative and inflammatory state triggered by a PHA antigen on RAW macrophages. These results are important because many studies have suggested that dysfunction of the innate immune system plays a role in the pathophysiology of BD whose symptoms can be controlled using Li. Patients with BD show high levels of proinflammatory cytokines. The innate immune system is a novel therapeutic target for treating BD, indicating that Li exerts important anti-inflammatory effects in patients with BD [26, 29].

Action of Li and LXC on levels of proinflammatory and anti-inflammatory cytokines and also cytokine and GSK-3 protein could be considered more relevant results found in the present study.

Before we mainly discuss the results found here, it is important to conduct some considerations about the relevance of investigations using chemical matrix elaborated from isolated molecules in standardized and controlled conditions. In fact, beverages such as coffee, green and black tea, yerba mate, and guarana are highly consumed in the world, for healthy people and patients that present some psychiatric condition. These beverages present some similarities in their chemical matrix, mainly by the presence of caffeine, other xanthine molecules, and some types of catechins [30–32]. Therefore, we opted to investigate the anti-inflammatory effect of isolated molecules and a chemical matrix containing caffeine, theobromine, and catechin (LXC).

As initial results observed more effective anti-inflammatory effect in cells LXC-treated than cells just treated with isolated molecules, we concentrated our investigations just in LXC treatment. This choice is partially corroborated with some previous studies that found more effective anti-inflammatory effect of full beverages than isolated molecules. This is the caffeine case that has been broadly used as pharmacological drug or supplement in energetic beverages. A recent systematic review about human consumption of coffee or caffeine on serum concentration of inflammatory markers, such as IL-6 and IL-10 cytokines, suggested that there is a predominant anti-inflammatory action of coffee compared to isolate caffeine [33]. Moreover, green tea lowering effect against inflammatory conditions has been described in the literature, although additional investigations also found anti-inflammatory properties from isolated catechin polyphenols found in this beverage [34, 35]. Therefore, the initial results found here that observed more effective effect of LXC treatment than caffeine, theobromine, and catechin isolated molecules is plausible. Moreover, for our best knowledge, construction of “LXC chemical matrix” to investigate the concomitant and synergic* in vitro *anti-inflammatory effect was not performed yet.

In fact, XC chemical admixture tested here was experimentally designed based in the guarana chemical matrix, and initial results suggested its anti-inflammatory effect (Figure 1(b)). We choose guarana to elaborate the chemical matrix tested here based on prior* in vivo* and* in vivo* investigation that described guarana lowering effect on IL-1*β*, IL-6, and TNF*α* levels. Guarana was also able to increase the levels of IL-10 anti-inflammatory cytokine. In the same study,* in vitro* assay performed with human PBMCs showed that guarana presented similar effect to resveratrol, quercetin, and ascorbic acid isolated molecules that have recognized anti-inflammatory action [12]. Therefore, the results described by Krewer et al. [12] subsidized the conduction of complementary protocols to test the effect of concomitant Li and XC exposure in macrophage-activated cells.

In fact, LXC-treated cells presented lower levels of IL-1*β*, IL-C, and TNF*α* levels and higher IL-10 levels when compared to PHA-treated cells and also when compared to untreated control cells. Moreover, our results suggested that LXC and Li downregulated genes of these molecules, suggesting its nutrigenomic effect in the production of proinflammatory and anti-inflammatory cytokines by macrophage cells. The LXC anti-inflammatory effect observed here could be triggered by two possible interactive ways: direct antioxidant action of LXC on macrophage cells and consequent decrease of inflammatory process or by genomic modulation of some molecules involved in the regulation of inflammatory response, such as GSK-3*β*. Although oxidative stress has been consistently reported in BP disorder and the study performed by Scola et al. [e] to suggest that this alteration could be modulated by polyphenols obtained from a* Vitis labrusca* extract, in the present investigation, LXC effect on prooxidant and antioxidant molecules was not so remarkable. Probably, these results are related to the fact that PHA-exposure did not trigger an extensive oxidative stress on macrophages.

On the other, hand macrophage-activated cells presented higher GSK-3*β* levels that was directly modulated by Li and LXC treatments. Li lowering effect on GSK-3*β* levels, was consistent with that reported previously [3, 27, 28]. Our Li plus XC potentialized the GSK-3*β* inhibitory modulation observed in cells just Li-treated. GSK-3*β* is an enzyme characterized as a crucial pleiotropic molecule to neural functions that is expressed throughout the brain involved in several cellular processes as metabolism, neurogenesis, synaptic plasticity, and apoptosis [36]. Because of its important role of neuronal function, GSK-3 dysregulation is suggested to contribute to the pathogenesis of numerous neurological disorders, including neuroinflammation and neurodegenerative diseases [3].

GSK-3 has two isoforms, namely, GSK-3*α* and GSK-3*β*. GSK-3*β* is abundantly expressed in the brain and is implicated in cytoskeletal organization and remodeling. Inhibition of GSK-3*β* is one of the most relevant effects of Li treatment and involves two interrelated but distinct mechanisms. First, Li indirectly prevents the binding of Mg^2+^ to the catalytic core of GSK-3*β* by inducing the phosphorylation of its serine 9 residue, thus inducing a conformational change in and inactivating GSK-3*β*. Second, Li modulates GSK-3*β* levels by decreasing the transcription of its encoding gene [33]. In the present study, we observed that the LXC mixture downregulated the expression of the GSK-3*β* gene irrespective of Li supplementation. A study by Kolnes et al. [34] reported that caffeine and theophylline, another xanthine present in coffee and guarana beverages, block the phosphorylation of GSK-3*β* at its serine 9 residue. Another study evaluated the protective effect of EGCG in H_2_O_2_-treated H9c2 rat cardiomyoblast model of myocardial ischemia injury. Pretreatment with EGCG or a GSK-3*β* inhibitor improved cell viability, suggesting that EGCG also modulates GSK-3*β* level [35]. Unfortunately, we are not able to find some prior investigation involving theobromine modulatory GSK-3*β* effects.

## 5. Conclusion

Despite the methodological constraints associated with* in vitro* studies, the present study indicates that concomitant supplementation with Li and XC molecules could potentialize anti-inflammatory Li effect by intensification of the following: GSK-3*β* inhibitory modulation, lowering effect on proinflammatory cytokines, and increase in the levels of IL-10 that is an anti-inflammatory cytokine. These results provide useful information for treating patients with BD and other psychiatric disorders because consumption of beverages containing caffeine, theobromine, and catechins, such as coffee, green and black tea, yerba mate, and guarana, is a part of the routine dietary pattern of a large number of people worldwide.
